# Sleep disturbances predict long-term changes in low back pain among Finnish firefighters: 13-year follow-up study

**DOI:** 10.1007/s00420-014-0968-z

**Published:** 2014-08-02

**Authors:** Sirpa Lusa, Helena Miranda, Ritva Luukkonen, Anne Punakallio

**Affiliations:** 1 Physical Work Capacity Team, Finnish Institute of Occupational Health, P.O. 486, 33101 Tampere, Finland; 2 Occupational Health Services, OP-Pohjola Group, Helsinki, Finland; 3 Statistical Services Team, Finnish Institute of Occupational Health, Helsinki, Finland; 4 Physical Work Capacity Team, Finnish Institute of Occupational Health, Helsinki, Finland

**Keywords:** Back disorders, Longitudinal studies, Sleep, Firefighters

## Abstract

**Purpose:**

To investigate the prevalence of low back pain among Finnish firefighters and to examine whether sleep disturbances predict membership of low back pain trajectories.

**Methods:**

In this prospective study, 360 actively working firefighters responded to a questionnaire in 1996, 1999 and 2009. The outcome variables were radiating and local low back pain during the preceding year. Using logistic regression modeling, the likelihood of membership of pain trajectories was predicted by sleep disturbances at baseline.

**Results:**

During the 13-year follow-up, the prevalence of radiating low back pain increased from 16 to 29 % (*p* < 0.0001) and that of local low back pain from 28 to 40 % (*p* < 0.001). The following trajectories were identified: “pain free,” “recovering,” “new pain,” “fluctuating” and “chronic.” More than one-fifth of the participants belonged to the new pain trajectory as regards both pain types, 6 % of the participants belonged to the chronic radiating and 12 % to the chronic local low back pain trajectory. Those with sleep disturbances at baseline had a 2.4-fold risk (adjusted OR 2.4; 95 % CI 1.2–4.7) of belonging to the new pain or chronic radiating pain cluster compared to pain-free participants.

**Conclusions:**

This is the first prospective study to show that low back symptoms are common and persistent among firefighters and that sleep disturbances strongly predict membership of a radiating pain trajectory. Occupational health and safety personnel, as well as the firefighters themselves, should recognize sleep problems early enough in order to prevent back pain and its development into chronic pain.

## Introduction

Firefighting is a universal profession, with rather similar work features across countries, i.e., extinguishing fires, performing rescue operations and often also medical first aid. Firefighting work has considerable physical as well as psychological demands, causing high loading of both the body and mind. However, firefighters’ health problems, especially musculoskeletal disorders, have rarely been reported in epidemiological studies. (Sluiter and Frings-Dresen 2007).

Early retirement due to disability is frequent among firefighters. In Finland, for example, little <70 % of operative firefighters are able to work until their normal retirement age (63‒68 years). In 2008‒2010, the mean age of disability retirement among Finnish firefighters’ was 53. The most common reasons for early retirement are musculoskeletal (43 %), mental (14 %) and cardiovascular (14 %) disorders. The most common medical diagnoses (16 % of all diagnoses) for early retirement are related to low back (e.g., degeneration of lumbar disk). (A Koski-Pirilä, The Local Government Pensions Institution, personal communication, 2011).

The number of full-time workers in the fire and rescue sector (including firefighters and paramedics/ambulance drivers) in Finland is approximately 5,000. In addition, about 14,000 part-time employees and voluntary fire brigade members are available for emergency situations (Ministry of the Interior of Finland 2006). Each year in Finland, some 85,000 emergency operations are carried out by firefighters, and this number has doubled over the last 10 years. In addition, approximately 200,000 urgent ambulance call-outs are also answered by firefighters in Finland each year (Ministry of the Interior of Finland 2006).

Most of the above-mentioned firefighting and rescue tasks require extremely good musculoskeletal health. Increasing problems in daily work tasks at fire stations, due to firefighters’ musculoskeletal problems, may occur among the aging workforce in particular. There is an obvious need to gain a better understanding of the long-term course of low back pain among firefighters and of factors affecting this. From occupational health personnel’s perspective, more information on symptoms and their risk factors is essential in order to better treat and prevent these problems. Firefighters would benefit from such information: They could learn what to expect in terms of symptoms, how pain may impact their work and why interventions may be needed.

Sleep problems are a potential risk factor in the development of pain symptoms among firefighters. In addition to physically strenuous work, firefighters are also exposed to abnormally long work shifts (often 24-h). A recent study showed that firefighters’ sleep problems reduce physical well-being (Carey et al. 2011). Sleep problems have also been established as a risk factor for pain, especially back pain, among workers other than firefighters. In a study among blue- and white-collar Finnish workers, sleep disturbances independently doubled the risk of developing low back pain during a 1-year follow-up (Miranda et al. 2008).

The aim of this 13-year follow-up study was to:

1. investigate the prevalence of radiating and local low back pain among Finnish firefighters at baseline and during the 3- and 13-year follow-up.
2. examine whether sleep disturbances at baseline predict the likelihood of belonging to a pain trajectory.

## Methods

### Study design

The data were based on a 3- and 13-year follow-up study of the health, and physical and mental capacity of Finnish professional firefighters (Lusa et al. 2006; Punakallio et al. 2012). The study consisted of repeated extensive questionnaires as well as objective measurements of the health and physical capacity of firefighters. This paper is based on these self-administered questionnaires. The study was approved by the Ethics Committee of the HUS Hospital District, and was performed according to the ethical principles of the Declaration of Helsinki.

At baseline in 1996 (T0), 1 124 participants out of 3 512 professional operative male firefighters were selected from all over Finland by stratified sampling (Punakallio et al. 1999). The baseline sample was representative of Finnish firefighters.

### Outcomes

#### Radiating and local low back pain

Information on radiating and local low back pain both at baseline and follow-ups was elicited using a question based on a validated Nordic questionnaire that has high repeatability and sensitivity (Kuorinka et al. 1987). The question was: “Estimate for how many days altogether you have had radiating (or local) low back pain during the last 12 months.”

The answers were classed into two categories: “0 = no pain” (pain on 0‒7 days or not at all), “1 = pain” (including pain on 8‒30 days, pain >30 days but not daily, or daily). Our study inquired about radiating and local low back pain separately, as have other previous studies (for example Miranda et al. 2002). We assumed that there is a set number of distinct courses of radiating and local low back pain and that the participants could be grouped into distinct trajectories, with each participant belonging to a certain trajectory.

### Predictors and covariates

The variables treated as predictors were chosen on the basis of the literature and pre-analysis of the data (correlation analysis of the predictors and outcome variables). The main predictor of interest was sleep disturbances, elicited through a self-administered questionnaire in 1996. Sleep disturbances were considered mild if the firefighter reported either not sleeping well during the last 3 months or having been extremely tired during the daytime for at least 3‒5 days a week; and severe if they reported both (Partinen and Gislason 1995). This measure has been used in many epidemiological studies (e.g., Jansson-Fröjmark and Lindblom 2008; Linton 2004), and is considered fairly reliable (e.g., Biering-Sørensen et al. 1994).

#### Covariates

The variables included as covariates in the analysis were as follows: age, pain other than low back pain, work accidents, smoking, physical workload and psychosocial job demands. Age was classified as <30, 30‒40 and >40 years. Pain other than low back pain, information on which was elicited by the Nordic Questionnaire (Kuorinka et al. 1987) (neck, shoulder, upper-arm, hip and knee), was classed into two categories: “0 = no pain” (pain on 0‒7 days or not at all), “1 = pain” (pain on 8‒30 days, pain >30 days but not daily, or daily) and a sum variable was formed. Work accidents were elicited by the question: “Over the last 3 years, have you suffered accidents or minor injuries at work? If so, how many?” Answers were categorized into 0, 1, 2 or >2. Smoking was inquired about by two different questions: “Have you ever smoked regularly?” (yes/no). “Do you still smoke?” (yes/no). We categorized the participants into never smokers, ex-smokers and current smokers. Physical workload was measured using four items adapted from Viikari-Juntura et al. (1996). The questions were as follows: “How many hours on average per shift do you work on your knees, on your hunches, squatting or crawling?” (1 = not at all, 2 < 1/2 h, 3 = 1/2‒1 h, 4 =>1 h), “How many hours on average per shift do you work with your back bent forward?” (1 = <1/2 h, 2 = 1/2‒1 h, 3 = 1‒2 h, 4 =>2 h) and “How much do you estimate that you work with your back twisted during a regular shift?” (1 = not at all, 2 = a little, 3 = moderately, 4 = a lot). A sum variable was formed from the items (3‒12) and categorized into three classes: <6, 6‒7 and > 7.

Psychosocial job demands consisted of four items based on and modified from the questions of earlier studies and the analysis by Airila et al. (2012): responsibility of job, fear of failure at work, excessive demands of work (Tuomi et al. 1991) and lack of supervisor’s support (Elo et al. 1992). Items were rated on a five-point scale (0 = none, 1 = few, 2 = some, 3 = rather many, 4 = very many). We formed a variable of the items (0‒16): none (0), few (1‒4), some (5‒8) and rather many/very many (9‒16). We also asked about work experience (“How many years have you been working in the rescue department?”) and working hours (“What kind of working hours do you do: 24-h shift work, other kind of shift work, regular daytime work, other?”).

### Data analysis

First, the prevalence of low back pain, the distribution of the participants into the different pain trajectories, and the characteristics of the trajectories were analyzed by applying cross-tabulations (chi-square tests) and *T* tests. Associations between variables were studied by Pearson’s and Spearman’s correlation analysis.

We tried to form trajectories by two-step cluster analysis, available in SPSS Statistics 17.0. In addition, we tried to identify trajectories using the modeling strategies available in statistical software package SAS version 9.2 (SAS Institute Inc. 2008). We also continued to form many kinds of pain course combinations for radiating and local low back pain according to our own hypothesis.

The likelihood of belonging to a certain pain trajectory was predicted by sleep disturbances at baseline using logistic regression modeling (proportional odds model). The models were formed so that in the first model only sleep disturbances were the predictor. Secondly, we added age to the model. Then, sleep disturbances adjusted by age and covariate formed their own separate models, one at a time. Finally, the last model was formed by backward stepwise logistic regression analysis. First, sleep disturbances and all the main covariates were entered into the same model. We continued by eliminating variables one at a time until all the remaining variables were significant at the critical level of 0.05. Odds ratios and their 95 % confidence intervals were calculated. In the outcome variable (pain trajectories), the reference group was those who belonged to the pain-free trajectory. The statistical analyses were carried out using the SAS statistical software package, version 9.2 (SAS Institute Inc. 2008).

## Results

### Participants

Altogether 849 (76 %), 794 (72 %) and 721 (68 %) firefighters answered in 1996 (T0), 1999 (T1) and 2009 (T2), respectively, after two reminders. Of the 2009 sample, 63 % (*n* = 451) were still working in the fire and rescue sector. The most common reasons for drop-out were old-age retirement (18 %, *n* = 125), disability pension (7 %, *n* = 48), change of job (4 %, *n* = 28) and sick leave (3 %, *n* = 23).

The sample of this study was formed from the participants who responded to each questionnaire and worked actively in firefighting and rescue tasks during the follow-up. The final sample comprised 360 male firefighters. Their mean age at baseline was 36 ± 5.4 years. The number of non-respondents after baseline was 465. They were older (41.6 ± 9.0) than the participants of this study (Table 1); more than half of them (59 %) were over 40 years of age. They had longer work experience, did shift work more often, and more often had mild or severe sleep problems and musculoskeletal pain other than back pain. They also seemed to have higher psychosocial job demands and were more often smokers. No differences were found in physical workload, work accidents or the prevalence of radiating or local low back pain compared to the respondents.

**Table 1:** Characteristics of follow-up cohort and non-respondents (retired/drop-outs)

Characteristics in 1996	Follow-up cohort (actively working participants, *n* = 360)	Retired or dropout due to non-response (*n* = 465)
Age (years), mean ± SD	35.7 ± 5.4	41.6 ± 9.0
Age group [*n* (%)]		
<30	46 (13)	60 (13)
30‒40	219 (61)	130 (28)
>40	95 (26)	275 (59)
Work experience (years) mean ± SD	12.3 ± 5.3	17.3 + 8.2
Working hours [*n* (%)]		
24-h shift work	265 (74)	375 (81)
Other kind of shift work	62 (17)	58 (13)
Regular daytime work	24 (7)	22 (5)
Other	8 (2)	7 (1)
Sleep disturbances [*n* (%)]		
None	208 (58)	235 (51)
Mild	137 (38)	194 (42)
Severe	14 (4)	33 (7)
Radiating low back pain [*n* (%)]	53 (16)	77 (19)
Local low back pain [*n* (%)]	95 (28)	111 (26)
Musculoskeletal pain in body parts other than back [*n* (%)]	207 (58)	265 (58)
Smoking [*n* (%)]		
Never smoker	74 (21)	75 (16)
Ex-smoker	117 (33)	112 (24)
Current smoker	168 (47)	277 (60)
Physical workload sum index (0–12) [*n* (%)]		
<6	121 (34)	132 (29)
6‒7	140 (39)	186 (42)
8‒12	97 (27)	129 (29)
Number of work accidents during last 3 years [*n* (%)]		
0	43 (20)	47 (19)
1	61 (28)	74 (29)
2	51 (24)	61 (24)
>2	60 (28)	71 (28)
Psychosocial job demands sum index (0‒16) [*n* (%)]		
None (0)	108 (30)	113 (24)
Few (1‒4)	193 (54)	226 (49)
Some (5‒8)	48 (13)	101 (22)
Many/very many (9‒16)	11 (3)	22 (5)

### Radiating and local low back pain

Table 2 shows the proportion of the participants who reported having had radiating pain in the low back on more than 7 days during the preceding 12 months. The prevalence of radiating low back pain increased during the 3-year follow-up from 16 to 23 % (*p* < 0.05) and rose during the 13-year follow-up to 29 % (*p* < 0.0001). The prevalence of local low back pain was higher than radiating low back pain at baseline (28 %) and increased significantly during the 13-year follow-up, reaching 40 % at the end of the follow-up.

**Table 2:** Prevalence of radiating and local low back pain of actively working firefighters in 1996, 1999 and 2009 (*n* = 360) and significant differences between years, *p*

Musculoskeletal pain	Prevalence						*p*	*p*
	1996		1999		2009		1996	1996
	%	*n*	%	*n*	%	*n*	1999	2009
Radiating low back pain	16	(53)	23	(76)	29	(100)	<0.05	<0.0001
Local low back pain	28	(95)	28	(95)	40	(137)	ns	<0.001

### Trajectories of radiating and local low back pain

**Fig. 1:**
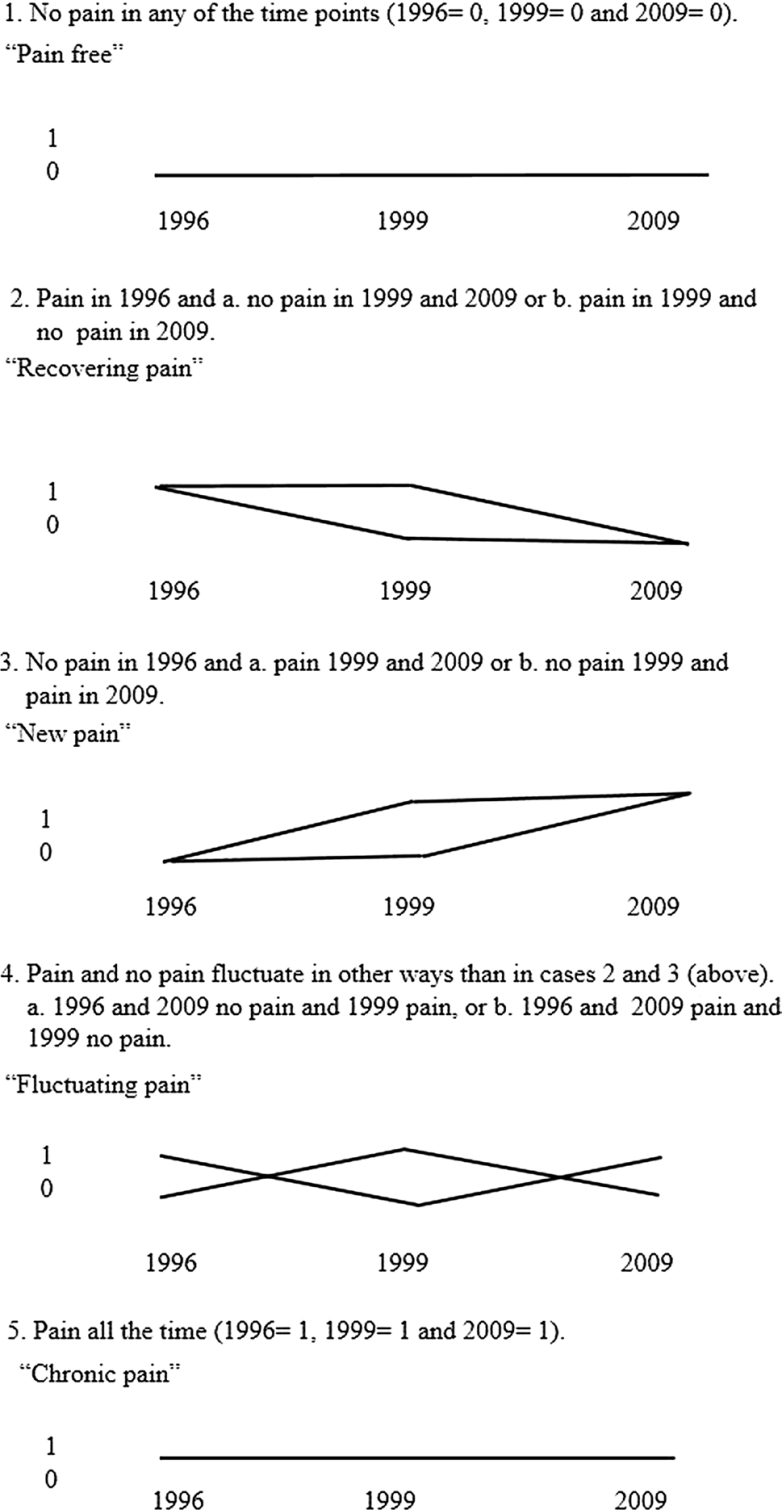
Description of the pain trajectories formed in this study

After meticulous analysis, we found five trajectories that best described the courses of radiating and local low back pain. These five trajectories, based on our own pre-analysis and hypothesis, were as follows: pain free, recovering, new pain, fluctuating and chronic (Fig. 1). We also formed five trajectories by the two-step cluster analysis available in SPSS Statistics 17.0, but in this sample they did not function as well as our own division of the trajectories. The main differences occurred in the cases in which the pain category changed during the follow-up time (recovering, new pain and fluctuating). The pain-free and chronic groups were the same in both analyses. The two-step cluster analysis also placed some of the cases of new pain and fluctuating pain, as well as recovering and fluctuating pain, together. In addition, the program automatically formed only four clusters, and we think that these clusters were problematic in the same way as described above. Therefore, we considered that our own trajectories best described the courses of pain during the 13-year follow-up. In the models, both outcome variables were categorized into three categories: 1: pain free, 2: recovering or fluctuating, 3: new pain or chronic. The reason for combining recovering and fluctuating into one category (in the analysis) is that at one study point at least, the participants (in this trajectory) were pain free. How this differed to the new pain and chronic trajectory is that the trend of the pain course was not so clear.

Many of the respondents belonged to the pain-free trajectory: of radiating low back pain more than half (54 %), and of local low back pain, 41 %. However, almost one-fourth (24 %) of the participants belonged to the new pain trajectory of local low back pain and about one-fifth (21 %) to the new pain trajectory of radiating pain. In the chronic pain trajectory, 6 % of the participants had radiating and 12 % of the participants had local low back pain. The proportions of the recovering trajectory were 8 % radiating and 11 % local low back pain (Table 3).

**Table 3:** Proportion of actively working firefighters belonging to different trajectories of radiating and local low back pain in 1996, 1999 and 2009 (*n* = 360)

Musculoskeletal pain	Trajectory									
	Pain free		Recovering		New pain		Fluctuating		Chronic	
	%	*n*	%	*n*	%	*n*	%	*n*	%	*n*
Radiating low back pain	54	(148)	8	(21)	21	(56)	11	(30)	6	(17)
Local low back pain	41	(126)	11	(33)	24	(73)	12	(35)	12	(36)

Table 4 shows the proportion of firefighters in each of the five radiating low back pain trajectories and their corresponding characteristics. The radiating low back pain trajectories did not differ significantly with respect to age, smoking and psychosocial job demands. In all trajectories, the majority of firefighters were 30‒40-year-olds at baseline. However, in the pain-free trajectory, one-fifth of firefighters were under 30, whereas in the chronic trajectory, 35 % were over 40.

**Table 4:** Characteristics of five trajectories of radiating low back pain and significant differences between trajectories, *p*

Characteristics in 1996	Pain free *n* = 148 (54 %)	Recovering *n* = 21 (8 %)	New pain *n* = 56 (21 %)	Fluctuating *n* = 30 (11 %)	Chronic *n* = 17 (6 %)	*p*
Age (years) mean ± SD	35 ± 6	37 ± 6	36 ± 5	37 ± 5	37 ± 4	
Age group [*n* (%)]						
<30	29 (20)	2 (10)	4 (7)	2 (7)	0 (0)	
30‒40	85 (57)	11 (52)	37 (66)	18 (60)	11 (65)	
>40	34 (23)	8 (38)	15 (27)	10 (33)	6 (35)	
Sleep disturbances [*n* (%)]						0.0044
None	96 (65)	9 (43)	20 (36)	3 (43)	8 (47)	
Mild	49 (33)	9 (43)	33 (59)	15 (50)	8 (47)	
Severe	3 (2)	3 (14)	3 (5)	2 (7)	1 (6)	
Musculoskeletal pain in other body parts [*n* (%)]	70 (56)	13 (81)	39 (83)	19 (79)	11 (85)	0.0013
Smoking [*n* (%)]						
Never smoker	40 (27)	5 (24)	8 (14)	4 (14)	1 (6)	
Ex-smoker	54 (36)	5 (24)	15 (27)	9 (31)	6 (35)	
Current smoker	54 (36)	11 (52)	33 (59)	16 (55)	10 (59)	
Physical workload sum index (0‒12) [*n* (%)]						0.007
<6	54 (37)	6 (29)	14 (25)	4 (13)	4 (24)	
6‒7	58 (39)	12 (57)	27 (49)	9 (30)	6 (35)	
8‒12	35 (24)	3 (14)	14 (25)	17 (57)	7 (41)	
Number of work accidents during last 3 years [*n* (%)]						0.002
0	22 (27)	4 (27)	5 (13)	0 (0)	2 (18)	
1	24 (30)	2 (13)	12 (30)	6 (27)	0 (0)	
2	24 (30)	3 (20)	10 (25)	7 (32)	1 (9)	
>2	11 (14)	6 (40)	13 (33)	9 (41)	8 (73)	
Job demands sum index (0‒16) [*n* (%)]						
None (0)	44 (30)	5 (24)	16 (29)	8 (27)	3 (18)	
Few (1‒4)	87 (59)	10 (48)	30 (54)	15 (50)	10 (59)	
Some (5‒8)	13 (9)	5 (24)	8 (14)	6 (20)	3 (18)	
Many/very many (9‒16)	4 (3)	1 (5)	2 (4)	1 (3)	1(6)	

Sleep disturbances at baseline seemed to be more prevalent in all the other trajectories except the pain-free trajectory of radiating low back pain (*p* = 0.0044) (Table 4). Musculoskeletal pain in other body parts at baseline seemed to be less common among firefighters belonging to the pain-free trajectory of radiating low back pain (*p* = 0.0013) than to the other trajectories. Moreover, there were fewer smokers (36 %) in the pain-free cluster. The proportion of smokers was highest in the new pain and chronic trajectory of radiating low back pain (59 and 54 %) (*p* = 0.0725) in 1996. Physical workload seemed to be highest in the fluctuating cluster (*p* = 0.007) and number of accidents in the chronic cluster (*p* = 0.002).

As regards local low back pain, the trajectories did not differ significantly from each other. The mean age of the firefighters in the chronic and fluctuating trajectory was lower (34 years) than that in the other trajectories (35‒37 years). It was also lower than the mean age of the chronic trajectory of radiating low back pain (37 years).

### Predictive models for membership of pain trajectories

Those firefighters who reported having sleep disturbances at baseline were three times more likely to belong to the new pain or chronic trajectory than to the pain-free trajectory of radiating low back pain (Table 5). The risk remained almost as high when the model was adjusted for age. Furthermore, after adding musculoskeletal pain in other body parts to the model, the risk was still 2.5-fold. Pain in other body parts (at baseline) also strongly predicted the risk of belonging to the new pain or chronic trajectory, OR 3.5 (CI 1.6–7.5), and to the fluctuating/recovering trajectory, OR 3.0 (CI 1.3–7.1). Sleep disturbances were also a strong predictor for belonging to the fluctuating/recovering cluster: They presented a 2.4-fold risk, and when adjusted for age, a 2.3-fold risk. The association was no longer significant after adjusting for pain in other body parts.

**Table 5:** Odds ratios (OR) and 95 % confidence intervals for predictor, predictor adjusted for age and adjusted for age and covariates one at a time, as well as final model, predicting membership of low back pain trajectory

Factors in 1996	Risk of belonging to trajectory OR (95 % CI)			
	Radiating low back pain		Local low back pain	
	Fluctuating/recovering versus pain free	New pain/chronic versus pain free	Fluctuating/recovering versus pain free	New pain/chronic versus pain free
Sleep disturbance	2.4 (1.3–4.7)	3.0 (1.7–5.3)	1.5 (0.8–2.7)	1.5 (0.9–2.5)
Adjusted by age				
Sleep disturbance	2.3 (1.2–4.4)	2.9 (1.6–5.1)	1.6 (0.9–3.0)	1.6 (0.9–2.7)
Sleep disturbance and musculoskeletal pain in other body parts	1.5 (0.7–3.2)	2.5 (1.3–4.9)	1.3 (0.6–2.7)	1.7 (0.9–3.1)
	3.0 (1.3–7.1)	3.5 (1.6–7.5)	1.4 (0.7–2.9)	1.7 (0.9–3.2)
Sleep disturbance and number of work accidents during last 3 years	2.5 (1.0–6.2)	2.1 (1.0–4.6)	1.2 (0.5–2.7)	1.2 (0.6–2.4)
	1.6 (1.1–2.5)	1.5 (1.1–2.2)	1.3 (0.9–1.9)	1.4 (1.0–2.0)
Sleep disturbance and smoking	2.1 (1.1–4.1)	2.7 (1.5–4.9)	1.6 (0.9–3.0)	1.5 (0.9–2.6)
	1.4 (0.9–2.2)	1.8 (1.2–2.6)	0.9 (0.6–1.3)	1.2 (0.9–1.8)
Sleep disturbance and physical work load	2.2 (1.1–4.2)	2.9 (1.6–5.2)	1.6 (0.8–2.9)	1.5 (0.9–2.6)
	1.7 (1.1–2.7)	1.3 (0.9–1.9)	1.0 (0.7–1.5)	1.2 (0.8–1.7)
Sleep disturbance and job demands	2.2 (1.1–4.2)	2.8 (1.6–5.1)	1.6 (0.9–3.0)	1.5 (0.9–2.6)
	1.2 (0.8–1.9)	1.1 (0.7–2.7)	1.0 (0.6–1.5)	1.1 (0.8–1.6)
Final model adjusted for age				
Sleep disturbance	1.5 (0.7–3.1)	2.4 (1.2–4.7)	0.4 (0.2–0.8)	0.5 (0.2–1.1)
Musculoskeletal pain in other body parts	3.2 (1.3–7.7)	3.8 (1.7–8.4)	0.3 (0.2–0.7)	1.0 (0.4–2.6)
Smoking	1.5 (0.9–2.4)	1.9 (1.2–2.9)	0.5 (0.3–0.9)	0.7 (0.4–1.3)

Logistic regression analysis, significant at the level of *p* < 0.05

After adjusting for sleep disturbances by age and other main covariates (work accidents, smoking, physical workload, job demands) one at a time, the risk of belonging to the new pain or chronic trajectory still remained over twice that of belonging to the pain-free trajectory, as did belonging to the fluctuating or recovering trajectory compared to membership of the pain-free trajectory.

In the final model, we added sleep disturbances and all co-factors (age, smoking, physical workload and psychosocial job demands) together. The predictive value of sleep disturbances remained significant. The risk of belonging to the new/chronic radiating low back pain trajectory was 2.4-fold (CI 1.2–4.7) compared to that of belonging to the pain-free trajectory. Musculoskeletal pain also remained a significant co-factor: OR 3.8 (CI 1.7–8.4) for new pain/chronic and OR: 3.2 (CI 1.3–7.7) for the fluctuating/recovering trajectory compared to the pain-free trajectory. In addition, smoking almost doubled the risk of new/chronic pain (CI 1.2–2.9).

We also formed the same models for local low back pain, but none of the chosen factors predicted belonging to the trajectories of local low back pain.

## Discussion

This study showed that low back pain is a common and persistent health problem among firefighters. Sleep disturbance was a strong predictor of persistent or onset of radiating low back pain. The development of local pain was not, however, affected by sleep.

We were able to establish five different trajectories of radiating and local low back pain during the 13-year follow-up: pain free, recovering, new pain, fluctuating and chronic.

Firefighters are a select group of professionals characterized by good physical fitness and health. Their fitness requirements are exceptionally high compared to those of many other professions due to the physically and mentally demanding work tasks related to firefighting. Somewhat unexpectedly, we found that a representative sample of actively working Finnish firefighters reported radiating and local low back pain as often as other Finnish male workers of corresponding age. Almost half (46 %) of the firefighters had radiating low back pain at some time point during the follow-up period. This is in line with the results of Heistaro et al. (2007), who found that 41 % of Finnish male workers have had radiating low back pain at some phase during their life.

Every fourth firefighter experienced new radiating low back pain and every fifth local low back pain during follow-up. Our results are, however, influenced by the healthy worker effect, i.e., selection bias due to disability retirement and dropout. It is likely that the reason for dropout or early retirement has in some cases been low back problems, since about one-fifth of the dropouts reported radiating and one-fourth local low back pain at baseline when they were still active in the workforce (Table 4). It is therefore likely that the true long-term prevalence of back pain among firefighters is considerably higher than that captured in our study and other similar types of prospective studies based of self-assessment.

However, due to the universal nature of firefighting, there is an emerging need for scientific studies on the health effects of the job. Only a few published studies exist on firefighters’ musculoskeletal disorders. Sluiter and Frings-Dresen (2007) reported that in the Netherlands, 20 % of firefighters younger than 25 reported low back complaints over a 6-month time period. Among firefighters aged 50‒54, the prevalence was 39 %. This age-related increase is in line with our results. In the Dutch study, those who reported having low back problems in addition to shoulder and knee problems, and who were older than 49, also reported decreased work ability due to these complaints. In another Dutch study by Bos et al. (2004), almost half (47 %) of Dutch firefighters (mean age 39 years) reported disabilities resulting from back complaints. Also in Finland, according to the statistics of the Local Government Pensions Institution, musculoskeletal problems (especially low back) are the most common reason for early retirement among Finnish firefighters. (A Koski-Pirilä, The Local Government Pensions Institution, personal communication, 2011).

We found five different trajectories of low back pain among Finnish firefighters: pain free, recovering, new, fluctuating and chronic musculoskeletal pain. With respect to radiating low back pain, these trajectories were statistically significantly distinguished by sleep disturbances, pain in other body parts, physical workload and work accidents. In the case of local low back pain, the factors did not distinguish the trajectories, which may be due to the non-specificity of this type of pain compared to radiating low back pain. Radiating low back pain is also a more severe type of pain than local low back pain.

The pathways of low back pain in primary care have been studied by Dunn et al. (2006). They concluded that their classification into four pathways of pain (“recovering,” “persistent mild,” “fluctuating” and “severe chronic”), by latent class analysis, provides a detailed alternative for improving understanding of the course of back pain. The pathways showed significant differences in disability, psychological status and work absence, and they were well maintained throughout a 1-year follow-up. Another study reported that most people remained in a similar trajectory in a 7-year follow-up (Wiesel 2011).

Tamcan et al. (2010) also investigated the course of low back pain in the general population using latent class analysis over a 1-year period. They identified four clusters of low back pain: “fluctuating,” “mild persistent,” “moderately persistent” and “severe persistent”; but did not have a “recovering” cluster in their study. Their four clusters differed significantly in relation to age and dependence on help. They also found that a considerable proportion of patients in the fluctuating group changed classifications. None of these studies investigated the predictors of group membership, as we did.

In earlier studies, pain pathways have been formed by latent cluster analysis and the studies have had various follow-ups, usually short in duration, i.e., 1 year (Dunn et al. 2006; Tamcan et al. 2010). In our study, the pain measurements and classifications were to a great extent different and the follow-up longer. Dunn et al. (2006) concluded that the optimal number of trajectories is either four or six for longitudinal latent class analysis. We also tried a two-step cluster analysis, which is available in SPSS, and this gave two different classifications: four and five clusters. However, they did not function as well as our own division of the clusters. The main differences were in the recovering, new and fluctuating trajectories, whereas the pain-free and chronic groups were the same. The two-step cluster analysis combined the cases of new and fluctuating, as well as recovering and fluctuating. We preferred to keep recovering separate; the numbers of participants in the recovering cluster was high enough (*n* = 22 % in radiating low back pain and 33 % in local low back pain). We considered our own clusters to better describe the course of the pain during the 13-year follow-up.

Many epidemiological studies have found that sleep disturbances increase the risk of further back pain and its development into chronic pain. Sleep problems also predict the need for hospital care, work disability, and pain in body parts other than the back (Eriksen et al. 2001; Hoogendoorn et al. 2001; Haig et al. 2006; Kaila-Kangas et al. 2006; Auvinen et al. 2010). Although there is evidence that pain leads to sleep disturbances, several studies also show that sleep disturbances may cause pain (for example Smith et al. 2009). For example, in a laboratory setting, it was found that the lack of REM-sleep in particular increased pain sensitivity (Lautenbacher et al. 2006; Roehrs et al. 2006). Possible mechanisms for the sleep–pain relationship are inflammation, changes in hormonal functions, metabolism and tissue regeneration (Lautenbacher et al. 2006; Roehrs et al. 2006). Sleep deprivation may also cause an increase in body weight, which in turn can lead to back pain. Sleep deprivation may also disturb the regulation of brain functions and increase chaos in the brain, affecting pain sensitivity (Irwin et al. 2006; Schmid et al. 2007).

In our study, sleep disturbances at baseline strongly predicted chronic or onset of radiating low back pain during the 13-year follow-up. The predictive power of sleep disturbances remained high after adjustment for age and further adjustment for physical workload and psychosocial job demands. Musculoskeletal pain in other body parts was a strong co-factor in the model. Since we have no information on the time before baseline, we cannot rule out the possibility that pain in body parts other than the low back may have preceded sleep disturbances. It is also possible that earlier back pain (before the first study) might have preceded sleep disturbances. There might also be reverse causality in the chronic trajectory, because participants in this group already suffered pain at baseline. Unfortunately, the number of participants did not allow us to study the predictive power of sleep disturbances in the baseline pain-free group or to compare it with that of the group with pain. Furthermore, we wanted to study the courses of pain. In our population, the predictive power of sleep disturbances remained significant after adjustment for shift work. This may be due to the fact that almost all the participants did shift work.

It is essential to understand the relationship between sleep disturbances and back pain, because many firefighters have sleep problems. In this sample of Finnish firefighters, 42 % reported sleep disturbances at baseline (and of the drop-outs 49 %). Many other studies on firefighters have indicated that sleep problems are also associated with other types of health problems, such as decreased psychosomatic well-being, vigilance, alertness and mental performance, as well as increased fatigue and depression (Lusa et al. 2002; Elliot and Kuehl 2007; Carey et al. 2011).

Among firefighters, sleep patterns may be disturbed by long work shifts and alarms. For example in Finland, the most common shift is the 24-h shift (Carey et al. 2011). The treatment of sleep problems in security occupations is challenging. The use of sleeping pills, for example, is not recommended due to the physically and mentally demanding nature of the work. For preventing sleep and other health-related problems early enough, environmental- and individual-based interventions should be planned for firefighters.

### Study strengths and limitations

The main strengths of our study lie in its longitudinal design. The 13-year study period with three measurement points allowed us to study the courses of pain over time and claim for at least some causality, although we could not completely exclude the possibility of reverse causality. We also had to take into account the fact that the periods between the study points were quite long (3 and 10 years), and we do not necessarily know all that happened during this time. At baseline, this study population was a representative sample of Finnish firefighters. The response rates to baseline and follow-up surveys were good. As we only included in this study the participants who responded on all three occasions, the number of dropouts was high. In addition to the health-based selection from the workforce, almost one-fifth of the dropouts retired normally on old-age pension, because of the low retirement age among Finnish firefighters during the study period, i.e., 55 years, and early retirement schemes and personal retirement arrangements (under 55 years of age) which are still possible routes for retirement. Therefore, dropout from the sample can be regarded as partly normal. However, our results are influenced by the healthy worker effect, which means that they are unlikely to overestimate the associations between sleep disturbances and low back pain.

This study was based on self-report measures, which may cause an overestimation of the associations between study variables due to common method variance bias. However, such bias is less likely in longitudinal studies (Doty and Glick, 1998). Furthermore, our data were mainly collected through widely used, valid and reliable questionnaires (Kuorinka et al. 1987; Tuomi et al. 1991; Elo et al. 1992; Linton 2004; Biering-Sørensen et al. 1994; Jansson-Fröjmark and Lindblom 2008). Information on symptoms was collected using the validated Nordic questionnaire, which is widely used, has high repeatability and sensitivity, and is considered an international standard (Kuorinka et al. 1987). A cutoff of above 7 days of pain and the 12-month time period of the follow-up are also commonly used criteria in musculoskeletal disorder studies (Miranda et al. 2002). In addition, the questions on sleep disturbances are widely used in epidemiological studies (Partinen and Gislason 1995; Miranda et al. 2008). They take into account both not sleeping well and tiredness after waking up. Most of the questions concerning the other covariates have been validated (Viikari-Juntura et al. 1996). A limitation concerning the questions was that the length of memory time varied.

Our study focused on only one profession and gender, male firefighters; and thus, the results can be generalized to other occupations and women only with caution. The sample at baseline, however, was comprehensively selected and was a good representation of Finnish firefighters.

## Conclusion

In conclusion, the results of this study help us better understand the different courses of back pain over a long time period. It also shows, for the first time among actively working firefighters, that sleep disturbances need to be taken into account in the prevention and treatment of back pain. In health examinations, musculoskeletal pain in all body parts should be monitored sufficiently early, together with sleep disturbances, so that the development of chronic pain could be prevented through individual-based or environmental interventions. Sleep guidance should be an essential part of workplace health promotion.
